# Stress ulcer prophylaxis in intensive care unit patients receiving enteral nutrition: a systematic review and meta-analysis

**DOI:** 10.1186/s13054-017-1937-1

**Published:** 2018-01-28

**Authors:** Hui-Bin Huang, Wei Jiang, Chun-Yao Wang, Han-Yu Qin, Bin Du

**Affiliations:** 1 Medical ICU, Peking Union Medical College Hospital, Peking Union Medical College and Chinese Academy of Medical Sciences, 1 Shuai Fu Yuan, Beijing, 100730 People’s Republic of China; 2 Department of Critical Care Medicine, the First Affiliated Hospital of Fujian Medical University, 20 Chazhong Road, Fuzhou, 350000 People’s Republic of China

**Keywords:** Stress ulcer prophylaxis, Enteral nutrition, Critically ill, Meta-analysis

## Abstract

**Background:**

Pharmacologic stress ulcer prophylaxis (SUP) is recommended in critically ill patients with high risk of stress-related gastrointestinal (GI) bleeding. However, as to patients receiving enteral feeding, the preventive effect of SUP is not well-known. Therefore, we performed a meta-analysis of randomized controlled trials (RCTs) to evaluate the effect of pharmacologic SUP in enterally fed patients on stress-related GI bleeding and other clinical outcomes.

**Methods:**

We searched PubMed, Embase, and the Cochrane database from inception through 30 Sep 2017. Eligible trials were RCTs comparing pharmacologic SUP to either placebo or no prophylaxis in enterally fed patients in the ICU. Results were expressed as risk ratio (RR) and mean difference (MD) with accompanying 95% confidence interval (CI). Heterogeneity, subgroup analysis, sensitivity analysis and publication bias were explored.

**Results:**

Seven studies (n = 889 patients) were included. There was no statistically significant difference in GI bleeding (RR 0.80; 95% CI, 0.49 to 1.31, *p* = 0.37) between groups. This finding was confirmed by further subgroup analyses and sensitivity analysis. In addition, SUP had no effect on overall mortality (RR 1.21; 95% CI, 0.94 to 1.56, *p* = 0.14), *Clostridium difficile* infection (RR 0.89; 95% CI, 0.25 to 3.19, *p* = 0.86), length of stay in the ICU (MD 0.04 days; 95% CI, −0.79 to 0.87, *p* = 0.92), duration of mechanical ventilation (MD −0.38 days; 95% CI, −1.48 to 0.72, *p* = 0.50), but was associated with an increased risk of hospital-acquired pneumonia (RR 1.53; 95% CI, 1.04 to 2.27; *p* = 0.03).

**Conclusions:**

Our results suggested that in patients receiving enteral feeding, pharmacologic SUP is not beneficial and combined interventions may even increase the risk of nosocomial pneumonia.

**Electronic supplementary material:**

The online version of this article (doi:10.1186/s13054-017-1937-1) contains supplementary material, which is available to authorized users.

## Background

Over the past decades, stress-related bleeding has become extremely uncommon in intensive care unit (ICU) patients [1]. Apart from pharmacologic approaches for stress ulcer prophylaxis (SUP), advances in the care of critically ill patients, such as optimal fluid resuscitation to maintain hemodynamic stability and thus improve splanchnic perfusion, and early provision of enteral nutrition (EN), may contribute to this observation [2–4]. Although recommended only in patients on mechanical ventilation or coagulopathy, patients with traumatic brain injury or major burns, or those with ≥ 2 risk factors [5, 6], SUP is still being used in nearly 90% of ICU patients, despite lack of an accepted indication in the majority [7–9]. Furthermore, SUP is often continued in these patients until clinical improvement, or even after transfer to the general ward [1, 10]. However, SUP is not without risks. The extensive use of SUP has been demonstrated to be associated with a higher rate of hospital-acquired pneumonia (HAP) due to loss of the protective bacteriostatic effect of gastric acid [4, 11]. Meanwhile, concomitant treatment of SUP and broad-spectrum antibiotics has also contributed to higher risks of *Clostridium difficile* infection [12, 13]. Thus, selection of potentially high-risk patients who may benefit from SUP while avoiding unnecessary use in others is important.

Some earlier studies reported that EN alone might provide sufficient prophylaxis against stress-related gastrointestinal (GI) bleeding [3, 14]. In animal models, enteral feeding is documented to increase GI blood flow and provide protection against GI bleeding [15, 16]. In a prospective, open-label trial, continuous EN was shown more likely than proton pump inhibitors (PPIs) or histamine 2 receptor antagonists (H_2_RAs) to raise gastric pH to above 3.5, suggesting that EN might be more effective in preventing GI bleeding than pharmacologic SUP [17]. Although several recent systematic reviews have comparatively evaluated pharmacologic agents for SUP, few of these studies have specialized in patients received EN [4, 18–20]. In 2010, one meta-analysis comparing H_2_RAs to placebo or no prophylaxis for SUP looked into a subgroup of enterally fed patients. In this subgroup, SUP did not decrease the risk of bleeding, and in contrast led to more episodes of hospital-acquired pneumonia (HAP) and higher mortality rate [4]. However, these findings were based on an evaluation of only 262 patients in three randomized controlled trials (RCTs) (three trials in GI bleeding, two trials in HAP and mortality), which were published between the years 1985 and 1994 and compared H_2_RAs with placebo [21–23]. In addition, two out of the three RCTs were unblinded [21, 22], and some of potentially important outcomes to clinicians or patients, including duration of mechanical ventilation, incidence of *C. difficile* infection, ventilator-associated pneumonia (VAP) and length of ICU stay were not considered in this meta-analysis.

Therefore, in order to address these limitations, we sought to expand the previous meta-analysis by adding relevant RCTs published between 1994 and 2017, and including any prophylaxis regimens. We reviewed these RCTs to determine if there are differences between pharmacologic SUP and placebo or no prophylaxis in enterally fed patients in terms of stress ulcer-related GI bleeding, and other clinical outcomes.

## Methods

### Search strategy and selection criteria

This systematic review and meta-analysis was conducted in accordance with the PRISMA guidance [24]. We searched RCTs in PubMed, Embase, and the Cochrane database from inception to 30 Sep 2017 to identify potentially relevant studies.

A population, intervention, comparator and outcomes assessment based on question and literature search was created (Additional file 1: S1). Our research was limited to RCTs and no language restriction was applied. Reference lists of included articles and other systematic review and meta-analysis were also reviewed. We included studies that met the following criteria: (1) design - RCTs; (2) population - adult (≥18 years old) ICU patients receiving EN; (3) intervention - patients receiving any pharmacologic SUP, regardless of dosage, frequency and duration; (4) control - patients receiving placebo or no prophylaxis; (5) predefined outcomes - GI bleeding, overall mortality at the longest available follow up, HAP, length of ICU stay, duration of mechanical ventilation and *C. difficile* infection. To facilitate comparison with the previous meta-analysis by Marik et al. [4], we required included studies to specifically report that > 50% of enrolled patients received EN [4]. We excluded studies enrolling patients who were < 18 years old, using SUP due to active bleeding or increased risk of bleeding, or receiving palliative care and publications available only in abstract form or meeting reports. Studies with inadequate information about enteral feeding were also excluded. We contacted the authors if the data on predefined outcomes from their studies were required.

### Data extraction and quality assessment

Two reviewers (H-BH and W J) independently extracted data from included studies, such as the first author, year of publication, country, sample size, study design, setting, treatment protocol for SUP and comparator, severity of illness, and all predefined outcomes. Quality of included studies was evaluated using the risk of bias tool recommended by the Cochrane Collaboration [25]. We assigned a value of high, unclear, or low to the following items: sequence generation; allocation concealment; blinding; incomplete outcome data; selective outcome reporting; and other sources of bias. Discrepancies were identified and resolved through discussion.

### Outcomes and statistical analysis

The primary outcome was bleeding rate, which was defined as overt GI bleeding (if reported in the enrolled studies) or clinically important GI bleeding (if overt GI bleeding was not reported in the enrolled studies). Secondary outcomes included incidence of HAP, overall mortality, *C. difficile* infection, length of ICU stay, and duration of mechanical ventilation. When the outcome of HAP was unavailable, the rate of VAP was used. The results from all relevant studies were merged to estimate the pooled risk ratio (RR) and associated 95% confidence intervals (CIs) for dichotomous outcomes. As to the continuous outcomes, mean difference (MD) and 95% CI was estimated as the effect result. Some studies reported the median as the measure of treatment effect, with accompanying interquartile range (IQR). Before data analysis, we estimated the mean from the median and standard deviation (SD) from the IQR using methods described in previous studies [26].

Heterogeneity was tested with *I*^2^ statistics. *I*^2^ < 50% was considered to indicate insignificant heterogeneity and a fixed-effect model was used, whereas a random-effect model was used in cases of significant heterogeneity (*I*^2^ > 50%). To explore the robustness and the potential influence of factors of our primary outcome, we performed subgroup analyses including type of SUP drugs (sucralfate, PPIs or H_2_RAs), route of administration (enteral or intravenous), study design (blinded or unblinded), sample size (<100 or >100), published year (before year 2000 or after year 2000), and clinical setting (medical, surgery, or mixed ICU). We also conducted sensitivity analyses on GI bleeding by pooling studies only focusing on: (a) overt GI bleeding; (b) clinically important GI bleeding; (c) a randomized-effects model; and (d) early EN (initiated within 48 hours of ICU admission). Publication bias was deemed to be evaluated by visually inspecting funnel plots when at least 10 studies were included in this meta-analysis. A *p* value <0.05 was considered statistically significant. All statistical analyses were performed using Review Manager, Version 5.3.

## Result

### Study selection

**Fig. 1:**
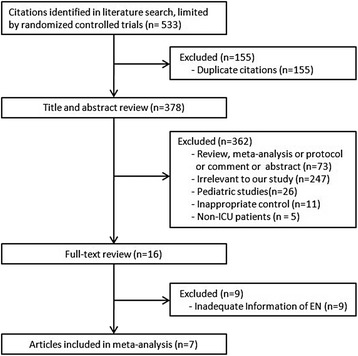
Selection process for randomized controlled trials (RCTs) included in the meta-analysis

A flowchart of the search strategy and the reasons for exclusion are shown in Fig. 1. The initial search identified a total of 533 citations: 155 studies were excluded because of duplicate studies, and 362 studies were excluded based on reviews of the title and abstract. Thus, 16 studies were full-text read for further evaluation. Of these 16 studies, 9 were excluded because they did not provide sufficient information on EN (Additional file 1: S2). Finally, the remaining seven RCTs, which enrolled 889 patients, were included in our final analysis [18, 21–23, 27–29].

### Study characteristics and quality

The main characteristics and predefined outcome data of the included RCTs are described in Tables 1 and 2. The variable definitions of GI bleeding and HAP are summarized in Additional file 1: file S3. These studies were published between 1985 and 2017, with sample sizes ranging from 28 to 300 patients. Four out of the seven RCTs used placebo as the comparator [18, 23, 27, 29], while the other three used no prophylaxis [21, 22, 28]. As for the type of prophylaxis drugs used, H_2_RAs and PPI were used in four [18, 27–29] and three studies [21–23], respectively, whereas H_2_RAs and sucralfate were used in one study [22]. During the study period, all patients received adequate EN (61–100%). Overt GI bleeding was reported in six RCTs [18, 21, 23, 27, 29], while three studies reported clinically important bleeding [18, 22, 28]. The Cochrane risk of bias score for each citation varied across the studies (Additional file 1: file S4). We did not assess the publication bias because of the limited number (<10) of studies included in each analysis.

**Table 1:** Characteristics of the included studies

Study/year	Sample size (I/C)	Setting	Prophylaxis drugs	Comparator	Patient characteristics (I/C)				
					Age, mean, (years)	Disease severity, median (IQR) or mean (SD)	Patient receiving EN, n/N (%)	MV (%)	Follow up (months)
Alhazzani et al. 2017 [18]	49/42	Mixed	Pantoprazole 40 mg once daily IV	Placebo	62/55	APACHE II score 21 (17–26)/22(14–27)	81/91 (89)	100/100	Unknown
El-Kersh et al. 2017 [29]	55/47	MICU	Pantoprazole 40 mg once daily IV	Placebo	62/58	SAPS II score 41 (34.5–53)/44 (34–54)	102/102 (100)	100/100	Unknown
Selvanderan et al. 2016 [27]	106/108	Mixed	Pantoprazole 40 mg once daily IV	Placebo	52/52	APACHE III score 66 (26)/66 (28)	214/214 (100)	100/100	12
Lin et al. 2016 [28]	60/60	Mixed	Lansoprazole OD 30 mg once daily	No prophylaxis	67/65	APACHE II score 21.3 (6.7)/19.9 (6.9)	120/120 (100)	100/100	1
Ben-menachem et al. 1994 [22]	200/100	MICU	Cimetidine 900 mg Infusion Sucralfate OD 1 g every 6 h	No prophylaxis	60/60	APACHE II score 17.4 (7.3)/16.5 (6.9)	198/300 (67)	74/65	10
Apte et al. 1992 [21]	16/18	MICU	Ranitidine 50 mg/every 6 h IV	No prophylaxis	27/26	MTS score 11 (4–16)/10 (6–16)	34/34 (100)	31/22	Unknown
Van den Berg et al. 1985 [23]	14/14	Mixed	Cimetidine 20 mg/kg/every 24 h IV	Placebo	44/48	-	17/28 (61)	100/100	Unknown

*APACHE II* acute physiology and chronic health evaluation II, *EN* enteral nutrition, *IQR* interquartile range, *I/C* intervention/control, *IV* intravenous, *MTS* maximum tetanus severity score, *MICU* medical intensive care unit, *Mixed* medical-surgical intensive care unit, *MV* mechanical ventilation, *OD* once daily, *SAPS II* simplified acute physiologic score II, *SD* standard deviation

**Table 2:** Predefined outcome of included studies

Study/year	GI bleeding		Mortality		Pneumonia		CDI		VAP		Duration of MV		Length of ICU stay	
	SUP	Control	SUP	Control	SUP	Control	SUP	Control	SUP	Control	SUP	Control	SUP	Control
Alhazzani 2017 [18]	4/49	3/42	17/49	13/42	10/49	6/42	2/49	1/42	10/49	6/42	9 (5–17)	6.5 (4–14)	12 (8–23)	8.5 (6–18)
El-Kersh 2017 [29]	1/55	1/47	7/55	8/47	-	-	1/55	3/47			4 (2.2–7)	5 (3–8)	6 (4–6.9)	7 (3.5–11.5)
Selvanderan 2016 [27]	3/106	6/108	30/106	25/108	12/106	8/108	1/106	0/108	12/106	8/108	21 (0–25)	21 (4–25)	6 (3–11)	7 (4–14)
Lin 2016 [28]	0/60	6/60	2/60	0/60	4/60	6/60	-	-	4/60	6/60	-	-	-	-
Ben-menachem 1994 [22]	10/200	6/200	45/200	19/100	25/200	6/100	-	-			7.3 (8.9)/8.1 (1.1)	7.9 (9.6)	3 (2–8.5)/4 (2–9)	3 (2–8)
Apte 1992 [21]	5/16	6/18	11/16	7/18	11/16	7/18	-	-			-	-	-	-
Van den Berg 1985 [23]	5/14	1/14	-	-	-	-	-	-			-	-	-	-

*CDI* Clostridium difficile infection, *GI* gastrointestinal, *MV* mechanical ventilation, *SUP* stress ulcer prophylaxis, *VAP* ventilator associated pneumonia

Continuous data are given as median (25th–75th percentile), mean (standard deviation, SD)

### Primary outcome

**Fig. 2:**
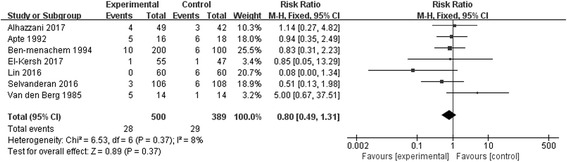
Forest plot showing the effect of stress ulcer prophylaxis for gastrointestinal bleeding. *M-H* Mantel-Haenszel

GI bleeding was reported in all seven RCTs. The pooled analysis showed that, in enterally fed patients, SUP did not reduce the risk of GI bleeding (7 studies; n = 889, RR 0.80; 95% CI, 0.49 to 1.31; *I*^2^ = 8%; *p* = 0.96) (Fig. 2). Although there was no significant heterogeneity, we proceeded to perform stratified analyses across predefined key study characteristics and clinical factors. In general, all the subgroup analyses confirmed similar rates of GI bleeding among groups. Sensitivity analyses were subsequently conducted, and suggested that when only clinically important GI bleeding or overt GI bleeding or randomized-effects models or early EN were considered, there was no difference between groups. Details of the results of subgroup analyses and sensitivity analyses are shown in Table 3.

**Table 3:** Further subgroup analysis and sensitivity analyses on primary outcome of gastrointestinal bleeding rate

		Studies, number	Patients, number	Event in SUP group	Event in control group	Risk ratio (95% CI)	*I* ^*2*^	*p*
Subgroup analyses								
Type of SUP	PPI	4	527	8 of 270	16 of 257	0.49 (0.21, 1.10)	4%	0.08
	H_2_RA	3	262	20 of 130	13 of 132	1.60 (0.86, 3.05)	16%	0.15
	Sucralfate	1	200	5 of 100	6 of 100	0.83 (0.26, 2.64)	-	0.76
Published year	After 2000	4	527	8 of 270	16 of 257	0.49 (0.21, 1.10)	4%	0.08
	Before 2000	3	362	20 of 230	13 of 132	0.75 (0.30, 1.86)	0%	0.53
Sample size	<100	3	153	14 of 79	10 of 74	1.42 (0.68, 2.94)	12%	0.35
	>100	4	736	14 of 421	19 of 315	0.52 (0.26, 1.04)	0%	0.07
Study designed	Blinded	4	435	13 of 224	11 of 211	1.12 (0.52, 2.44)	0%	0.77
	Unblinded	3	454	15 of 276	18 of 178	0.62 (0.32, 1.19)	35%	0.15
Setting	MICU	3	436	16 of 271	13 of 165	0.87 (0.44, 1.73)	0%	0.70
	Mixed ICU	4	453	12 of 229	16 of 224	0.73 (0.36, 1.50)	0%	0.73
Administration route	Oral	2	669	23 of 340	23 of 329	1.0 (0.58, 1.72)	0%	1.00
	Intravenous	6	320	5 of 160	12 of 160	0.35 (0.03, 3.84)	62%	0.39
Sensitivity analyses								
GI bleeding	Overt GI bleeding	6	589	18 of 300	23 of 289	0.79 (0.44, 1.39)	24%	0.41
	Clinical important GI bleeding	4	725	13 of 415	13 of 310	0.63 (0.29, 1.37)	25%	0.25
	Randomized-effects models	7	889	28 of 500	29 of 389	0.87 (0.50, 1.53)	8%	0.63
	Early enteral nutrition	6	798	24 of 451	26 of 347	0.76 (0.49, 1.29)	22%	0.31

*CIB* clinical important bleeding, *SUP* stress ulcer prophylaxis, *H*_*2*_*RA* histamine 2 receptor antagonist, *GI* gastrointestinal, *MICU* medical intensive care unit, *Mixed* medical-surgical intensive care unit, *PPI* proton pump inhibitor

### Secondary outcomes

**Fig. 3:**
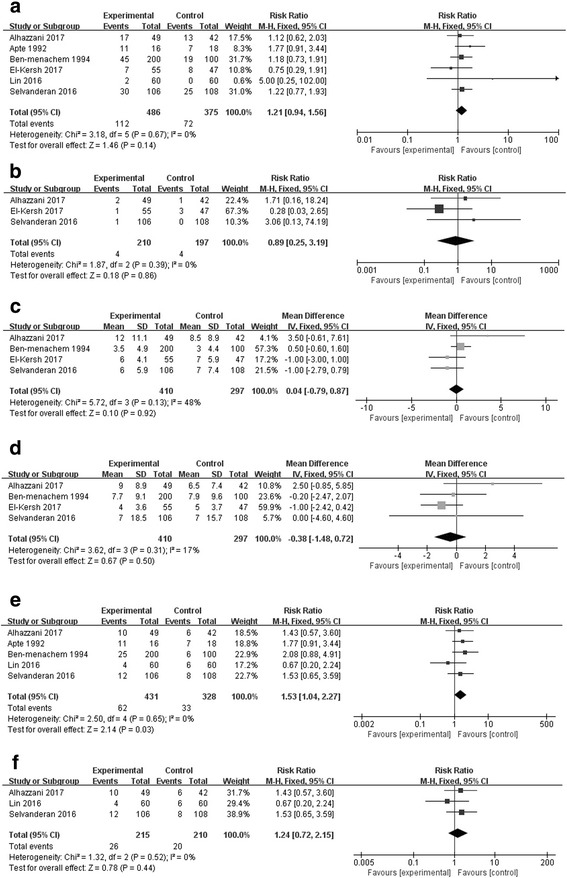
Forest plot showing the effect of stress ulcer prophylaxis on overall mortality (**a**), *Clostridium difficile* infection (**b**), length of intensive care unit stay (**c**), duration of mechanical ventilation (**d**), hospital-acquired pneumonia (**e**) and ventilator-associated pneumonia (**f**) *M-H* Mantel-Haenszel

There was no statistically significant difference between the SUP and the no SUP groups in overall mortality (6 studies, n = 861; RR 1.21; 95% CI, 0.94 to 1.56; *I*^2^ = 0%; *p* = 0.14) [18, 21, 22, 27–29] (Fig. 3a) or *C. difficile* infection (3 studies, n = 407; RR 0.89; 95% CI, 0.29 to 3.19; *I*^*2*^ = 0%; *p* = 0.86) [18, 27, 29] (Fig. 3b). The length of stay in the ICU (4 studies, n = 707, MD 0.04 days; 95% CI, −0.79 to 0.87, *I*^2^ = 48%; *p* = 0.92) [18, 22, 27, 29] (Fig. 3c) and duration of mechanical ventilation (4 studies, n = 707, MD −0.38 days; 95% CI, −1.48 to 0.72, *I*^2^ = 17%; *p* = 0.50) [18, 22, 27, 29] (Fig. 3d) were also similar. The incidence of HAP was higher in SUP group (5 studies, n = 407; RR 1.53; 95% CI, 1.04 to 2.27; *I*^2^ = 0%; *p* = 0.03) [18, 21, 22, 27, 28] (Fig. 3e), while the incidence of VAP was comparable between groups (3 studies, n = 425; RR 1.24; 95% CI, 0.72 to 2.15; *I*^2^ = 0%; *p* = 0.44) (Fig. 3f) [18, 27, 28].

## Discussion

Our meta-analysis showed that, among ICU patients receiving enteral feeding, pharmacologic SUP exerted no impact on the risk of GI bleeding, overall mortality, *C. difficile* infection, duration of MV and length of ICU stay, but led to an increased risk of HAP.

In this updated meta-analysis, we found that there was no added benefit with concomitant pharmacologic SUP in GI bleeding once patients were receiving enteral feeding. This finding expanded on the earlier meta-analyses to provide better evidence for pharmacologic SUP in enterally fed patients in the ICU [4]. First, our meta-analysis had a larger sample size than the previous meta-analyses as it included four RCTs published between 2016 and 2017, with more power to assess this effect. Second, the subgroup and sensitivity analyses based on various clinical characteristics did not significantly alter our main findings. Finally, we further evaluated other related important outcomes (e.g., duration of mechanical ventilation, overall mortality, and length of stay in the ICU) and found no difference between groups, thus providing evidence of the robustness of our results.

Apart from calorie delivery, EN had also been found to protect against stress-related GI bleeding [4, 14–16]. EN might mitigate macroscopic ulceration by optimizing mucosal energy, intramucosal pH [17, 30] and regional distribution of gastrointestinal blood flow [31, 32]. In addition, EN was able to reduce GI inflammation secondary to reperfusion injury. To date, there had been no RCTs comparing GI bleeding rates in critically ill patients receiving EN versus no EN. Several case series involving a total of 749 patients reported that enterally fed critically ill patients in the absence of pharmacologic SUP was associated with bleeding rates of 8.4–8.8% [3, 33]. In our study, we also found a similar bleeding rate (7.5%) in patients receiving EN alone. In comparison, Marik et al. reported a GI bleeding rate of 15.8% in the subgroup of patients without EN or any pharmacologic SUP [4]. A similar incidence (17.5%) was also identified in a recent meta-analysis in unfed patients [19]. These data suggest a potential role of EN against stress-related GI bleeding, therefore questioning any added benefits of pharmacologic SUP in patients already receiving EN.

Our results indicated that pharmacologic SUP in enterally fed patients is associated with higher incidence of HAP, which is consistent with the previous meta-analysis [4]. The reason might be that concomitant EN and pharmacologic SUP would result in a significantly higher pH than either intervention alone [17]. However, we should interpret this finding cautiously. First, the definitions of HAP varied across included studies, with the incidence ranging from 8.4 to 52.9% [18, 21–23, 27–29]. Second, the included studies had spanned a period of more than three decades, when co-interventions had been developed and quality improvement approaches such as guidelines for HAP/VAP prevention had been introduced and updated [34]. This, to some extent, might affect the accurate evaluation of the effects of SUP. In fact, the significant increase in pneumonia was mainly caused by the two earlier trials [21, 22], but not the newly published RCTs [18, 27, 28]. When only the three RCTs that focused on VAP were considered, no differences were found between groups [18, 27, 28]. Finally, we did not find significant differences in terms of other secondary outcomes (e.g., mortality, duration of mechanical ventilation, or length of stay in the ICU). Recently, more and more attention had been paid to the possible association between the SUP strategy and enteric infections, particularly *C. difficile* [13]. Several epidemiological investigations and meta-analyses had demonstrated an increased risk of *C. difficile* infection in patients under a SUP strategy. In addition, studies suggested that PPIs were more strongly associated with this enteric infection than H_2_RAs [12, 13]. However, these results could not place sufficient weight on RCT evidence. It was noteworthy that no study had investigated the effect of EN on *C. difficile* infection. Our results suggested that in enterally fed patients the rate of *C. difficile* infection was similar in the SUP and non-SUP groups (1.9% vs. 2.0%). The relatively small number of events may account for these negative results. Therefore, further well-designed, large RCTs are warranted to focus on this topic, as the detrimental outcomes of these serious infections may outweigh the benefit of SUP.

Recently, there has been growing interest in PPIs as a means of SUP. For example, all four additional RCTs included in our meta-analysis compared the effect of PPIs with placebo. Moreover, PPIs were also increasingly prescribed as the primary SUP agent, ranging from 39.6 to 70% in critically ill patients [8, 35]. In a recent international survey, PPIs were the most comment agent (66%) used for SUP [1]. This might be due to the superiority of PPIs in reducing GI bleeding, as suggested by several meta-analyses [20, 36], and the recommendations of the Surviving Sepsis Campaign [37]. Despite the widespread use of PPIs, the effect of concomitant EN in this SUP procedure is rarely evaluated. In our study, only four included RCTs focused on this topic, and pooled results suggested no benefit or harm associated with PPIs. Though limited by the small sample, our data may, at the very least, encourage clinicians to reevaluate their practice in prescribing prophylactic PPIs in critically ill patients. As a matter of fact, several ongoing RCTs comparing PPIs with placebo in ICU patients with high risk of GI bleeding may provide more convincing evidence in the future [38–40].

Our study has some limitations. First, only seven studies were included in our analysis, and most of them had a sample size of less than 200 [18, 21, 23, 28, 29], which would more likely result in overestimation of effect size. Thus, further studies in large cohorts are needed to validate our findings. Second, there were differences among included trials with regards to the adopted definition of GI bleeding, timing and duration of EN, and patient intolerance of EN, which might lead to the observed heterogeneity, and therefore compromise the robustness of our findings. Third, the uneven distribution of different underlying diseases among included studies might also exert a prognostic value. We planned to perform subgroup analyses to explore studies based on such diversities, which was hampered by nsufficient data. Fourth, although predefined subgroup analyses had been performed, some results of subgroups should be interpreted with caution due to small number of patients. Finally, we had not pre-published this updated meta-analysis protocol in a registry.

## Conclusion

In summary, based on available data, our results demonstrate that in ICU patients receiving EN, pharmacologic SUP offered no beneficial effect on the incidence of GI bleeding and other clinically important outcomes. Large-scale, well-designed RCTs will be needed to confirm our findings.

### Key messages

- In patients receiving EN in the ICU, pharmacologic SUP showed no beneficial effect on GI bleeding, overall mortality, *Clostridium difficile* infection, length of stay in the ICU or duration of mechanical ventilation, but was associated with an increased incidence of HAP.
- Further larger adequately powered RCTs with rigorous definitions and designs are warranted to confirm our results.

## Additional file

Additional file 1: **S1.** PICO question. **S2.** Excluded RCTs that did not provide sufficient information on EN. **S3.** Definitions of GI bleeding and nosocomial pneumonia in the included RCTs. **S4.** Risk of bias graph and summary of the included RCTs. (DOCX 39 kb)

## Abbreviations

CIs: Confidence intervals
EN: Enteral nutrition
GI: Gastrointestinal
H_2_RAs: Histamine 2 receptor antagonists
HAP: Hospital-acquired pneumonia
ICU: Intensive care unit
IQR: Interquartile range
MD: Mean difference
PPIs: Proton pump inhibitors
RCTs: Randomized controlled trials
RR: risk ratio
SD: Standard deviation
SUP: Stress ulcer prophylaxis
VAP: Ventilator-associated pneumonia
