# Supplementary material for: Stress ulcer prophylaxis in intensive care unit patients receiving enteral nutrition: a systematic review and meta-analysis
Source: Crit Care. 2018 Jan 28;22:20. doi: 10.1186/s13054-017-1937-1 (PMC5787340; doi:10.1186/s13054-017-1937-1)
Supplement: Additional file 1: — S1. PICO question. S2. Excluded RCTs that did not provide sufficient information on EN. S3. Definitions of GI bleeding and nosocomial pneumonia in the included RCTs. S4. Risk of bias graph and summary of the included RCTs. (DOCX 39 kb) [file 13054_2017_1937_MOESM1_ESM.docx]

**Stress ulcer prophylaxis in intensive care unit patients receiving enteral nutrition:**

**A systematic review and meta-analysis**

Hui-Bin Huang MD^1, 2*^, Wei Jiang MD^1*^,

Chun-Yao Wang MD^1^, Han-Yu Qin MD^1^, Bin Du MD^1^

*^1^Medical ICU, Peking Union Medical College Hospital, Peking Union Medical College and Chinese Academy of Medical Sciences, Peking, China*

*^2^Department of Critical Care Medicine, the First Affiliated Hospital of Fujian Medical University, Fuzhou, China*

**Corresponding author:**

Bin Du, MD

Peking Union Medical College Hospital,

Peking Union Medical College and Chinese Academy of Medical Sciences

Peking, China

1 Shuai Fu Yuan, Beijing, 100730, P R China

[dubin98@gmail.com](mailto:dubin98@gmail.com)

**Additional Files**

**Additional file S1:** PICO question

**Additional file S2:** Excluded RCTs that did not provide sufficient information of EN

**Additional file S3:** Definitions of GI bleeding and nosocomial pneumonia of the included RCTs

**Additional file S4:** Risk of bias graph and summary of the included RCTs

**Additional file S1**

**PICO question**

**Population:** intensive care OR critically ill OR critical care

**Intervention:** stress ulcer prophylaxis OR sup OR sucralfate OR h2ra OR h2rb OR h2ras OR h2rbs OR histamine 2 receptor antagonist OR histamine-2 receptor blocker OR nizatidine OR famotidine OR cimetidine OR ranitidine OR ppi OR OR ppis OR proton pump inhibitor OR pantoprazole OR omeprazole OR rabeprazole OR dexlansoprazole OR esomeprazole OR lansoprazole

**Comparator**: control OR placebo

**Outcomes**: gi bleeding OR gastrointestinal bleeding OR mortality OR death OR pneumonia OR vap OR ventilator-associated pneumonia OR nosocomial pneumonia OR hap OR hospital-acquired pneumonia OR cdi OR clostridium difficile infection OR duration of mechanical ventilation OR length of stay OR los

**Additional file S2**

**Nine excluded RCTs that did not provide sufficient information of EN:**

1. McAlhany JC, Czaja AJ, Pruitt B. Antacid control of complications from acute gastroduodenal disease after burns. J Trauma 1976; 16:645-649.

2. Halloran LG, Zfass AM, Gayle WE, Wheeler CB, Miller JD (1980) Prevention of acute gastrointestinal

complications after severe head injury: a controlled trial of Cimetidine prophylaxis. Am J Surg 139(1):44-48

0002-9610(80)90228-7.

3. Peura DA, Johnson LF (1985) Cimetidine for prevention and treatment of gastroduodenal mucosal lesions in patients in an intensive care unit. Ann Intern Med 103(2):173-177.

4. Moscona R, Kaufman T, Jacobs R, Hirshowitz B. Prevention of gastrointestinal bleeding in burns: the effects of cimetidine or antacids combined with early enteral feeding. Burns 1985; 12:65-67.

5. Rath T, Walzer LR, Meissl G. Preventative measures for stress ulcers in burn patients. Burns 1988;14: 504-507.

6. Reusser P, Gyr K, Scheidegger D, Buchmann B, Buser M, Zimmerli W (1990) Prospective endoscopic study of stress erosions and ulcers in critically ill neurosurgical patients: current incidence and effect of acid-reducing prophylaxis. Crit Care Med 18(3):270-274.

7. Simms HH, DeMaria E, McDonald L, Peterson D, Robinson A, Burchard KW. Role of gastric colonization in the development of pneumonia in critically ill trauma patients: results of a prospective randomized study. J Trauma 1991; 31: 531-537.

8. Martin LF, Booth FV, Karlstadt RG, Silverstein JH, Jacobs DM, Hampsey J, Bowman SC, D’Ambrosio CA, Rockhold FW (1993) Continuous intravenous cimetidine decreases stressrelated upper gastrointestinal hemorrhage without promoting pneumonia. Crit Care Med 21(1):19-30.

9. Cook D, Guyatt G, Marshall JC, Leasa D, Fuller H, Hall R, et al. A comparison of sucralfate and ranitidine for the prevention of upper gastrointestinal bleeding patients requiring mechanical ventilation. N Engl J Med 1998;338: 791-797.

**Additional file S3**

**Definitions of GI bleeding and nosocomial pneumonia of the included RCTs**

| Study/Year | GI bleeding | nosocomial pneumonia |
| --- | --- | --- |
| Alhazzani 2017 | **Clinically important GI bleeding**, defined as the presence of **overt GI bleeding** (i.e., hematemesis, frank blood or coffee ground nasogastric aspirate, melena, or hematochezia) plus one of these features in the absence of other causes: a spontaneous drop of systolic or diastolic blood pressure of greater than or equal to 20 mm Hg within 24 hours of upper GI bleeding, an orthostatic increase in pulse rate of greater than or equal to 20 beats/min and a decrease in systolic blood pressure of greater than or equal to 10 mm Hg, a decrease in hemoglobin of greater than or equal to 2 g/dL (20 g/L) in 24 hours or transfusion of greater than or equal to two units of packed RBCs within 24 hours of bleeding (17). Gastroduodenoscopy, angiography, and other interventions were at the ICU team’s discretion | **VAP** was diagnosed when there is a new or progressive radiographic infiltrate developed with no other obvious cause and the presence of any two of the following: 1) fever (temperature > 38°C) or hypothermia (temperature < 36°C), 2) relative neutropenia (< 3.0 × 106/L) or leukocytosis  (> 10 × 106/L) |
| El-Kersh 2017 | **Overt GI bleeding** was defined by the presence of coffee-ground aspirate in nasogastric tube or coffee-ground emesis, bloody secretions in nasogastric tube or hematemesis, melena or hematochezia. **Significant GI bleeding** was defined by a 3-point decrease in hematocrit within a 24-hour period with clinical signs of overt GI bleeding, or by an unexplained 6-point decrease in hematocrit in a 48-hour  period. | NA |
| Selvanderan 2016 | **Clinically significant gastrointestinal bleeding** was defined as an episode of **overt bleeding** (hematemesis, bloody gastric aspirate, melena, or hematochezia), accompanied by at least one of the following: 1) a reduction in mean arterial blood pressure of more than or equal to 20 mm Hg within 24 hours in the absence of another cause, 2) a reduction in hemoglobin of more than or equal to 20 g/L within 24 hours, or 3) a need for endoscopy or surgery to achieve hemostasis. | We used the Centers for Disease Control and Prevention (CDC) definitions for infective **ventilator-associated complications and pneumonia** |
| Lin 2016 | **Apparent UGI bleeding** within 2 weeks of enrollment, which was defined as follows: (1) a coffee ground substance from the NG aspirate >60 mL; (2) fresh blood from the NG tube; or (3) passage of tarry stool. Secondary end points included **clinically significant UGI bleeding** (definition: UGI  bleeding with hemoglobin level decrease >2 gm/dL or in need of a blood transfusion of >2 units). | **Ventilator-associated pneumonia** (definition: clinical  pulmonary infection score21 >6, a scoring system composed of body temperature, white blood cell count, purulent secretions, diffuse or localized pulmonary infiltrate in CXR, progression of infiltrate, and culture of secretions) |
| Ben-menachem 1994 | **Substantial gastrointestinal hemorrhage** required the presence of any of the following: 1) persistent hematemesis (red blood or guaiac-positive "coffee grounds" that did not clear with 1.5 L saline lavage; 2) 3-point decrease in hematocrit during 24 hours accompanied by red blood or guaiac-positive "coffee grounds" material that cleared with lavage, or melena, or three guaiacpositive stools without evidence of lower gastrointestinal bleed; and 3) any unexplained 6-point decrease in hematocrit during a 48-hour period. This last criterion was added as a safety measure because some patients would not receive prophylaxis. | Diagnosis of **nosocomial pneumonia** in the medical ICU required all of the following: 1) chest roentgenogram obtained 72 hours or more after ICU admission that showed a new and persistent infiltrate; 2) fever, leukocytosis, or both; 3) purulent tracheobronchial secretions; 4) Gram-stained sputum showing more than 25 polymorphonuclear leukocytes and fewer than 10 squamous epithelial cells per low-power field; and 5) recovery of an accepted nosocomial pathogen from sputum culture. |
| Apte 1992 | **Gross bleeding** (bright red or altered blood), and examined once every morning for occult bleeding (benzidine test). | **Pneumonia** defined as the appearance of new infiltrates on the chest radiograph or branchial breath sounds on examination and a positive tracheal culture along with fever (axillary temperature >38℃),leukocytosis (>13,000 cells/mm3), and purulent sputum (>25 leukocytes per low-power field). All criteria were essential for diagnosis. |
| Van den Berg 1985 | **Blood loss** from the upper gastrointestinal tract was measured by labeling the erythrocytes in 10 ml of autologous blood with 25 uCi of ^51^Chromium chloride. These labeled erythrocytes were re-injected intravenously at the beginning of the treatment period and the gastric contents were aspirated either continuously or hourly, starting the first day of treatment.  **Blood loss** was calculated from the radioactivity in the gastric contents. Previous experience in a group of about 30 intensive care patients, whose gastric contents were collected by the same technique, showed that these patients lost between 1 and 7 ml of blood per 24 h. Blood loss greater than double the highest figure, 15 ml/24 h. was therefore considered to be suggestive of mucosal damage. | NA |

**Additional file S4**


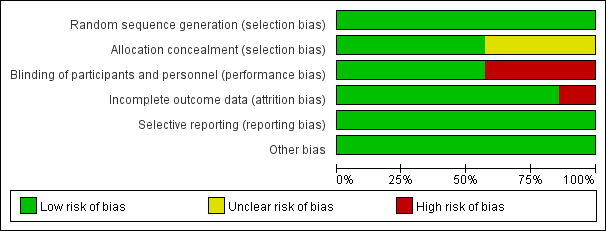


Risk of bias graph of the included RCTs. Red=high risk; green=low risk; yellow=unclear


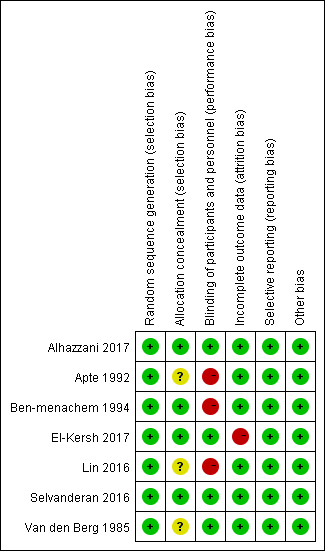


Risk of bias summary of the included RCTs
